# Transcranial Direct Current Stimulation Enhances Exercise Performance: A Mini Review of the Underlying Mechanisms

**DOI:** 10.3389/fnrgo.2022.841911

**Published:** 2022-04-26

**Authors:** Shapour Jaberzadeh, Maryam Zoghi

**Affiliations:** ^1^Non-invasive Brain Stimulation and Neuroplasticity Laboratory, Department of Physiotherapy, School of Primary and Allied Health Care, Faculty of Medicine, Nursing and Health Science, Monash University, Melbourne, VIC, Australia; ^2^Discipline of Physiotherapy, School of Health, Federation University Australia, Churchill, VIC, Australia

**Keywords:** exercise performance, transcranial direct current stimulation (tDCS), central fatigue, mental fatigue, peripheral fatigue, endurance exercise, strength exercise

## Abstract

Exercise performance (EP) is affected by a combination of factors including physical, physiological, and psychological factors. This includes factors such as peripheral, central, and mental fatigue, external peripheral factors such as pain and temperature, and psychological factors such as motivation and self-confidence. During the last century, numerous studies from different fields of research were carried out to improve EP by modifying these factors. During the last two decades, the focus of research has been mainly moved toward the brain as a dynamic ever-changing organ and the ways changes in this organ may lead to improvements in physical performance. Development of centrally-acting performance modifiers such as level of motivation or sleep deprivation and the emergence of novel non-invasive brain stimulation (NIBS) techniques such as transcranial magnetic stimulation (TMS) and transcranial direct current stimulation (tDCS) are the key motives behind this move. This article includes three sections. Section Introduction provides an overview of the mechanisms behind the reduction of EP. The main focus of the Effects of tDCS on EP section is to provide a brief description of the effects of tDCS on maximal and submaximal types of exercise and finally, the section Mechanisms Behind the Effects of tDCS on EP provides description of the mechanisms behind the effects of tDCS on EP.

## Introduction

Enhancing exercise performance (EP) portrays the everyday goal for many healthy young persons. In the context of sports, athletes are forced to push their bodily limits to run or swim quicker, lift heavier weights, perform some tasks better or jump higher or further. Therefore, competitors from all different athletic events are encouraged to use innovative approaches to increase their performance. EP is affected by a combination of factors including physical, physiological, and psychological factors. During the last century, numerous researches from different fields of studies were carried out to increase physical performance by modifying these factors (Schubert and Astorino, 2013). Therefore, much of the modern-day literature has ignored the significance of the brain in the regulation of physical performance.

Introduction and development of new non-invasive techniques such as neuroimaging techniques and non-invasive brain stimulation techniques, shed light on the role of the central nervous system on human performance during exercise. As the first step, neuroimaging techniques such as functional magnetic resonance imaging (fMRI) shed light on the role of specific brain areas during simple tasks involving a group of muscles in a single joint or multiple groups of muscles during a more complex task involving multiple parts of the body.

Furthermore, non-invasive brain stimulation techniques (NIBS) such as transcranial direct current stimulation (tDCS) or the centrally-acting performance modifiers, i.e. level of motivation or sleep deprivation are used to modulate these brain areas. incidentally, emerging literature indicates the possibility of influencing performance outcomes following stimulation of specific brain areas. Therefore, during the last two decades, the focus of research has been mainly moved toward the brain as a dynamic ever-changing organ and the ways changes in this organ may lead to improvements in EP.

Altogether, research studies on the use of tDCS provide remarkable understandings about the possible mechanisms behind the effects of tDCS on cortical neurons which finally led to enhancement of EP in healthy individuals. TDCS generates low intensity electric field within the brain (Datta et al., 2009; Edwards et al., 2013; Stagg et al., 2018; Truong and Bikson, 2018). There are two related mechanisms of tDCS that support its use for improvement of EP. The first mechanism is modulation of neuronal excitability and the second one is plasticity (Jackson et al., 2016). Traditionally, it has been established in animal and human studies that application of tDCS can induce polarity-specific changes in neuronal excitability. According to these studies “anodal” tDCS depolarizes neurons and increases neuronal firing frequency and “cathodal” tDCS hyperpolarizes neurons and decreases their firing rates (Creutzfeldt et al., 1962) (**Figure 2**). It should be noted that, this notion is an oversimplification. The whole neuron does not uniformly depolarize or hyperpolarize in response to the polarity of the applied current. Instead, every neuron has a number of compartments which some of them are depolarizing and others are simultaneously hyperpolarized during application of direct currents (Radman et al., 2009; Rahman et al., 2013). Indeed, the compartments nearer the cathode hyperpolarizing and the ones nearer the anode depolarizing. The polarization in these compartments will be reverses by changes in the polarity of the stimulation. Evidence also supports the non-linear dose-response relationships between tDCS application and the induced changes in corticospinal excitability. A number of recent studies indicate that anodal tDCS may also reduce or cathodal tDCS may also increase the corticospinal excitability (Batsikadze et al., 2013; Monte-Silva et al., 2013; Lopez-Alonso et al., 2014; Tremblay et al., 2016).

When the length of tDCS application increases and passes several minutes, both animal (Bindman et al., 1964; Reato et al., 2015) and human studies using TMS (Nitsche and Paulus, 2000) have confirmed changes in neuronal excitability that remains for minutes or hours after termination of stimulation. Animal models have further linked long lasting variations in excitability of the cortical/brain areas with synaptic plasticity. Long-term potentiation (LTP) and depression (LTD) are examples of these changes (Gartside, 1968; Kronberg et al., 2017; Yu et al., 2019). LTP is a process by which synaptic connections between neurons become stronger with frequent activation (Bliss and Cooke, 2011). On the other hand, LTD is a process by which synaptic connections between neurons become weaker with frequent activation (Bliss and Cooke, 2011). The changes in brain excitability, measured during or immediately after tDCS, and plasticity based on indicators of LTP or LTD are related.

The main objectives of this review are: to provide an overview of different models of fatigue as the underlying mechanisms behind the reduction of EP, a brief description of the effects of tDCS on maximal and submaximal types of exercise, and finally a description of the mechanisms behind the effects of tDCS on EP.

## The Mechanisms Behind the Reduction of EP

Fatigue could be considered as factor for reduction of EP. Traditionally, different discipline of sport science provided different definition for fatigue. These definitions are developed to best suit the individual disciplines. For example, an expert in biomechanics may define fatigue as a reduction in the force output generated by a muscle (Allman and Rice, 2002; Millet et al., 2003) and an expert in psychology may define fatigue as a “sensation” of tiredness (Kayser, 2003) on the other hand an expert in physiology may describe fatigue as the malfunction of a specific physiological system (Green, 1997).

Our ambiguity about the understanding of fatigue is may be related to the viewpoint that fatigue is an unfortunate event which due to some involuntary peripheral physiological or biochemical factors reduces EP (Damasio et al., 2000; Noakes and St Clair Gibson, 2004; St Clair Gibson and Noakes, 2004; Lambert et al., 2005; Datta et al., 2009; Dantzer et al., 2014). It may also relate to the use of a reductionist or cause-and-effect approach by the experts from different disciplines of sport which tried to shed light on different underlying mechanisms behind fatigue (St Clair Gibson and Noakes, 2004). Consequently, several direct cause-and-effect models have been introduced to describe fatigue (Noakes, 2000). These models include:

### Cardiovascular/Anaerobic Model

In this model, cardiovascular system failure in delivery of oxygen and removal of waste products to and from the active muscles is considered as the main reason behind the fatigue (Noakes, 2000; Noakes et al., 2001).

### Energy Supply/Energy Depletion Model

In this model, failure to supply sufficient ATP via different metabolic pathways to the active muscles (Noakes, 2000; Allman and Rice, 2002) is considered as the main reason behind the fatigue.

### Neuromuscular Model

In this model, the factors affecting muscle excitation, recruitment and contraction are considered as the main factors behind the fatigue (Noakes, 2000; Millet et al., 2002).

### Muscle Trauma Model

This model suggests that muscle damage due to prolonged muscle activity may led to a reduction in capacity of the active muscles to produce power (Gollhofer et al., 1987; del Aguila et al., 1999).

### Biomechanical Model

This model suggests that efficiency of movement patterns during muscle activity is main reason behind the fatigue. A more efficient movement pattern will lead to: drop the required VO_2_ (Gissane et al., 1991), to reduce energy storage (Hahn and Gore, 2001), to hinder the build-up of the metabolites and finally reduces the rise of core body temperature.

### Thermoregulatory Model

This model suggests that a critical core body temperature is a main reason behind the reduction or termination of the exercise Coyle and Montain, 1992; Kay et al., 1999).

### Psychological/Motivational Model

In this model a lack of enthusiasm or interest in EP is considered as the main factor for reduction or termination of muscle performance (Hargreaves and Febbraio, 1998; Nybo and Nielsen, 2001). This model is usually merged with the neuromuscular model of fatigue.

### Central Governor Model

In this model, the main assumption is that the attenuation or termination of EP is controlled by a constant feedback system (Noakes et al., 2001). This system includes a central controller which receive afferent somatosensory pathways and send information on force, displacement, time and muscular metabolism toward the active muscles.

### Complex System Model

**Figure 1:**
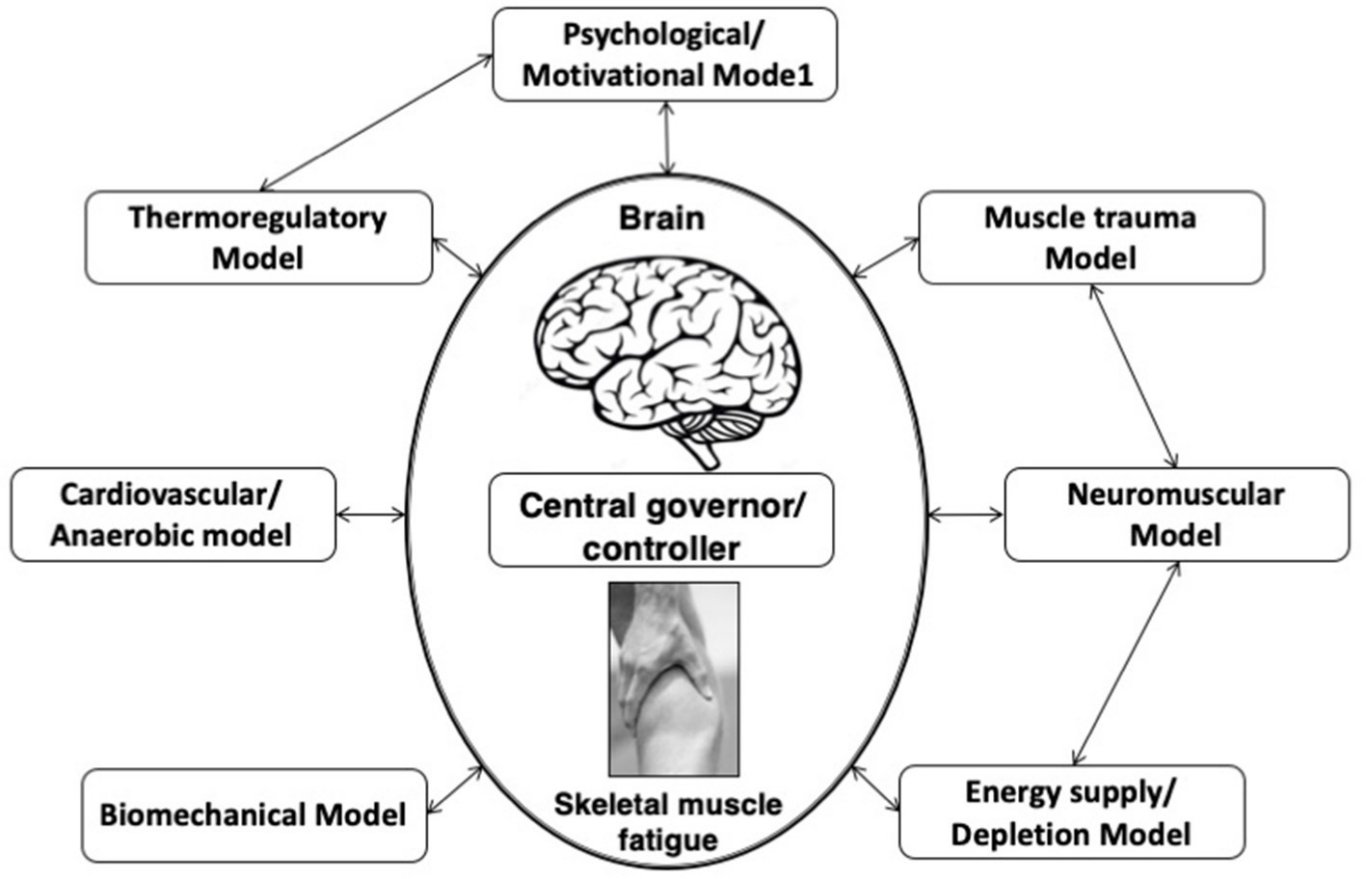
Complex systems model of fatigue. Interaction of developed fatigue models. In this complex systems model of fatigue peripheral feedback originating from numerous linear models of fatigue are integrated by the brain, along with centrally located senses.

This model is an extension of the central governor model (Figure 1) and postulated that skeletal muscle fatigue is not influenced by any one of the aforementioned single linear models. According to this model, EP is continuously controlled by the interaction of multiple physiological systems checked by continuous feed-forward and feedback mechanisms (St Clair Gibson and Noakes, 2004; Lambert et al., 2005).

**Figure 2:**
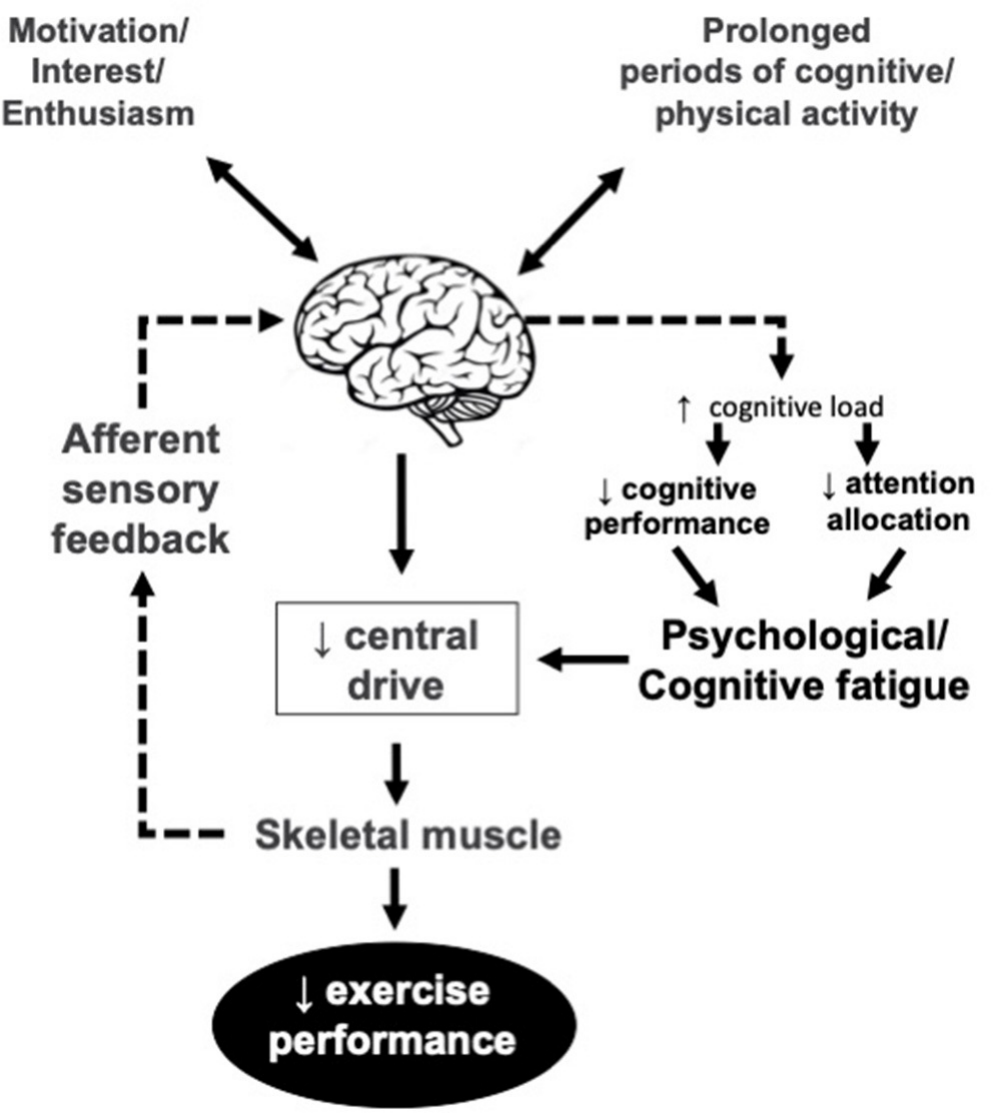
Psychological/mental model of fatigue. Central drive is reduced due to lower motivation, interest and/or enthusiasm and lack of cognitive resources for the EP. The reduced enthusiasm may or may not be related to afferent sensory feedback.

The idea of the central nervous system as contributing factor in the development of fatigue is back to the early work by Mosso (1904) which concluded a decreased capacity to perform repeated muscle contractions after a prolonged period of demanding cognitive activity, lead to the development of a psychobiological state which is called psychological or mental model of fatigue (Figure 2). It has implications for many activities of daily living including physical performance.

Psychological or mental model of fatigue is related to lack of energy, motivation, and alertness increased fatigability, and feelings of tiredness (Dantzer et al., 2014), and a reason behind the increased risk of error in the workplace (McCormick et al., 2012). From a neurophysiological viewpoint, mental fatigue inhibits athletes' performance by enhancing perceived exertion (Ishii et al., 2014) and also by deactivation of the mechanisms responsible for neurofacilitation that normally encourages athletes toward action (Hallett, 2007). It decreases physical performance even during long-duration exercise which seems less dependent on cognitive functioning (Marcora et al., 2009; Elferink-Gemser and Hettinga, 2017; Van Cutsem et al., 2017). Unlike endurance exercise, mental fatigue seems not to affect athletes' maximal strength, explosive power, and anaerobic work (Boksem et al., 2006; Dantzer et al., 2014; Martin et al., 2015; Van Cutsem et al., 2017).

Different fatigue mechanisms and adaptation of power outputs in long and short duration tasks is the reason behind this difference (Gandevia, 2001). Short duration, anaerobic types of exercises are mainly affected by peripheral fatigue (Coggan and Coyle, 1991), while long duration, aerobic (endurance) types of exercises, are affected by a decline in central motor drive and central fatigue (Amann, 2011). Hence, while during endurance exercise, the brain decides when to stop, during short-term exercise, the muscles are the main decision-makers (Gandevia, 2001). In this regard, Mental fatigue may disrupt the decision-making process involved in choosing an optimal pacing strategy (Martin et al., 2018).

## The Effects of tDCS on EP

**Figure 3:**
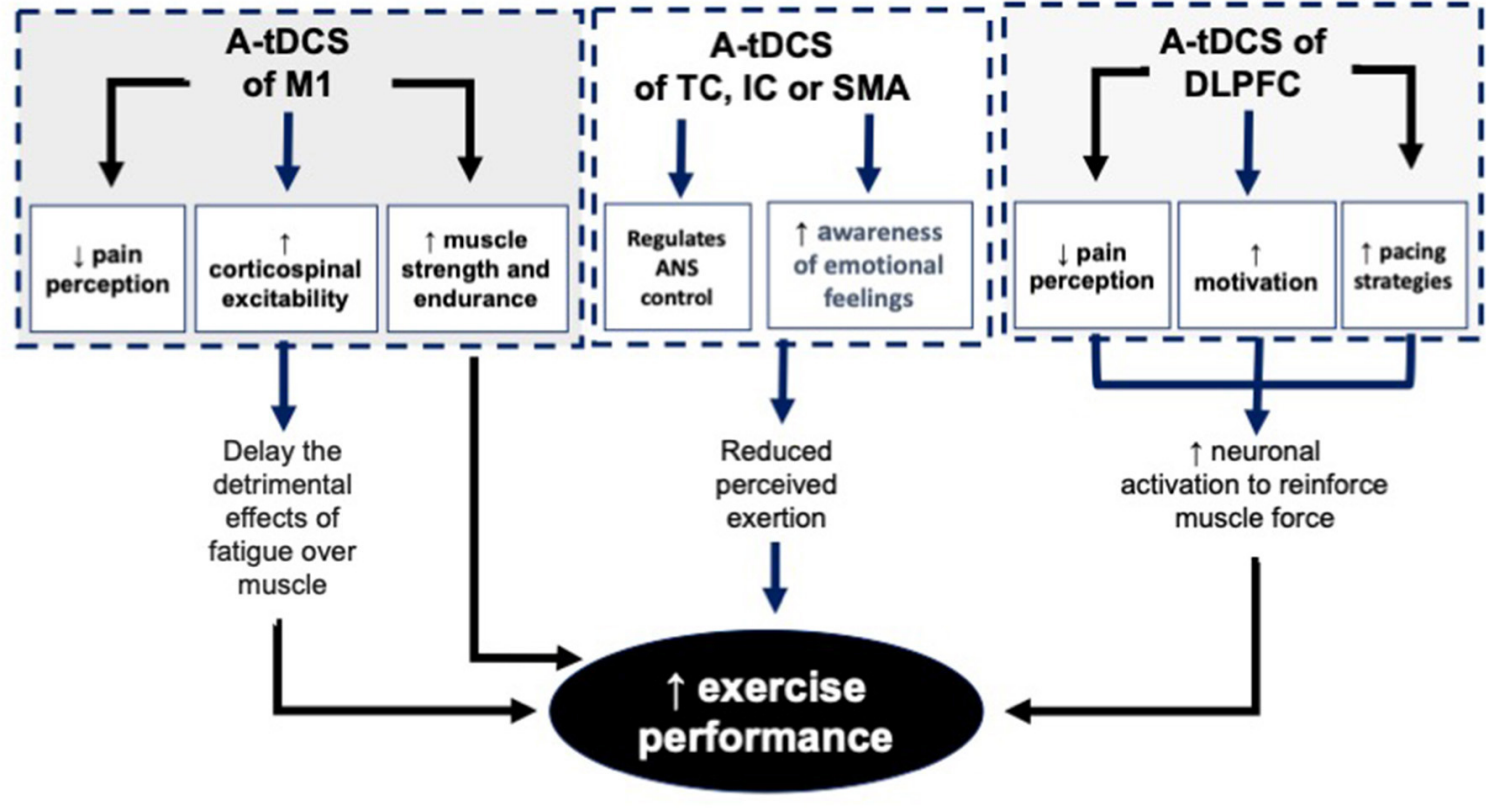
The mechanisms behind the effects of A-tDCS of M1, TC, IC, SMA and DLPFC on EP. A-tDCS, Anodal transcranial direct current stimulation; M1, Primary motor cortex; TC, Temporal cortex; IC, Insular cortex; SMA, Supplementary motor area; DLPFC, Dorsolateral.

The literature on EP provides exciting understandings on the effects of tDCS on EP in healthy individuals (Figure 3). In these studies, the effects of tDCS were investigated on maximal (explosive) and submaximal (endurance) performances. This is important because these different exercise intensities require completely different metabolic, cardiorespiratory, and psychological demands, and therefore affect the brain activity differently (Sidhu et al., 2013).

### How tDCS of M1 Affects Maximal Force Capacities

Despite the methodological differences in study design, experimental tasks, tDCS parameters, and montages in different studies, the literature indicates that a single session of unilateral tDCS over the dominant M1 failed to improve anaerobic maximal types of exercise (Cogiamanian et al., 2007; Kan et al., 2013; Williams et al., 2013; Angius et al., 2015, 2016a,b; Baldari and Buzzachera, 2018; Romero-Arenas et al., 2021). da Silva Machado et al. (2021), in a single session tDCS study compared the effects of conventional tDCS of M1 (2 mA, 20 min) with high definition tDCS (2.4 mA, 20 min) on exercise performance (time to exhaustion at 80% peak power) on a cycle simulator. They concluded, a single session of neither HD-tDCS nor conventional tDCS changed exercise performance and psychophysiological responses in athletes.

The reason behind this failure may lie in the fact that under maximal force conditions, muscles are already functioning maximally and all motor units within the involved muscles are already recruited and therefore the ceiling effects do not allow tDCS to show any further effects.

However, unlike the findings in these studies, recently, Lattari et al. (2020) concluded that 2 mA, 20 min bilateral tDCS of M1 significantly increases power during power-related tasks such as the vertical jumping ability. Codella et al. (2021), is also showed that bilateral tDCs of M1, enhance power in lower limb muscles.

### How tDCS of M1 Affects Submaximal Force Capacities

Having said that, the same literature (listed in the previous section) indicates positive effects of a single session of tDCS on submaximal intensity tasks in the majority of the studies (Cogiamanian et al., 2007; Williams et al., 2013; Angius et al., 2015, 2016a,b, 2018; Vitor-Costa Okuno et al., 2015; Abdelmoula et al., 2016; Lattari et al., 2018; Huang et al., 2019; Codella et al., 2021).

### How tDCS of DLPFC or TC Affects EP

The number of tDCS studies on other cortical sites of the brain such as dorsolateral prefrontal cortex (DLPFC) or temporal cortex (TC) is very low.

To investigate the effect of a single session tDCS of DLPFC (2 mA for 15 min) on the force-velocity relationship, strength training volume, movement velocity, and PRE in healthy non-professional participants, Alix-Fages et al. (2020), showed an increase in training volume, preservation of higher movement velocities, and reduction of ratings of perceived exertion (RPE) values. Lattari et al. (2018) applied tDCS over the left DLPFC (2 mA for 20 min) before a time to exhaustion (TTE) test (100% of peak power) in 11 moderately active women and found longer TTE compared to sham. Angius et al. (2019), used a similar protocol, and applied a single session of tDCS over the left DLPFC (2 mA) but with a longer duration (30 min) in 12 trained participants before a TTE test (70% of peak power). Participants were able to cycle for longer durations after tDCS, with lower HR and RPE compared to sham.

It is important to note that despite the positive results previously reported, some studies, however, have found no improvement using relatively similar protocols to the previous studies. Holgado et al. (2019) (*n* = 36), investigated the effects of tDCS of DLPFC (2 mA for 20 min) on power output, heart rate, RPE, and electroencephalography at baseline and during a 20-min time-trial self-paced exercise. They concluded neither power output, heart rate, RPE nor electroencephalography activity were affected by tDCS.

Similarly, the two studies on the effects of tDCS of TC on EP, are also concluded opposite conflicting findings on EP. Evidence shows that a single session tDCS (20 min, 2 mA) of TC, targeting the left insular cortex (IC), enhanced cycling performance in professional cyclists. They also showed that tDCS of TC decreases heart rate and increased delay in parasympathetic vagal withdrawal and RPE in submaximal exercise intensities (Okano et al., 2015). Unlike the positive findings in the above study, Okano et al. (2017) evaluated the effects of a single session tDCS of left TC targeting the left IC (20 min, 2 mA), on physiological and psychological responses during 30 min of vigorous exercise with a constant load (80% heart rate). The findings of this study suggest that tCDS of TC does not modulate either heart rate at rest or heart rate, RPE, and affective responses during exercise.

### How Direct Current Stimulation (DCS) of the Spinal Cord Affect EP

Sasada et al. (2017), investigated the effects of a single session of direct current stimulation (DCS) of the spinal cord (2 mA for 20 min) using the Halo Sport device on physical fitness indicators of healthy, physically active, men (*n* = 17). All participants underwent either stimulation or sham, before a vertical jump, sit & reach, and endurance running tests. The results suggest that DCS of spinal cord using a Halo Sport system can enhance the output in these three physical fitness measures in physically active participants.

### How Bilateral tDCS of M1 Using a Halo Sport Device Affect EP

The Halo Sport device is a commercial brain stimulation device that contains a headset like a usual headphone. This device uses weak direct currents below 2–3 mA (tDCS) which can be applied bilaterally over the scalp through surface electrodes. The main objective of this application is the induction of changes in motor cortex in both sides of the brain.

Huang et al. (2019), investigated the effects of a single session bilateral tDCS of M1 (2 mA for 20 min) using the Halo Sport device on repeated sprint cycling ability (*n* = 9). Peak and mean power output were measured for 5 × 6-s sprints interspersed with 24 s of active recovery on a cycle ergometer. The results suggest that tDCS with the Halo Sport system can enhance mean power output in physically active participants. In another study, Codella et al. (2021), investigated the effects of a single session bilateral tDCS of M1 (2 mA for 20 min) using the Halo Sport device on physical fitness indicators of healthy, physically active, men (*n* = 17). All participants underwent either stimulation or sham, before a vertical jump, sit and reach, and endurance running tests. The results suggest that tDCS with the Halo Sport system can enhance the output in these three physical fitness measures in physically active participants.

## Mechanisms Behind the Effects of tDCS on EP

The underlying mechanisms behind the positive effects of tDCS of M1 on EP are not fully understood yet. Literature indicates the following mechanisms behind the effects of a-tDCS on the enhancement of EP:

### Facilitation of M1 and Enhancement of Corticospinal Excitability

TDCS depends on the parameters used, may facilitate the M1, and therefore enhancing corticospinal excitability during exercise (Cogiamanian et al., 2007; Williams et al., 2013). This hypothesis is challenged by Abdelmoula et al. (2016) which concluded lack of relationship between the improvement in corticospinal excitability and EP. It should be noted that the M1 is not the only active brain site during exercise, therefore well-designed double-blinded studies to establish the relationship between the cortical/corticospinal changes and EP is necessary.

### Reduction of the Fatigue Through Affecting the Central Governor/Controller

TDCS of M1 may also lead to a reduction of fatigue (Cogiamanian et al., 2007; Williams et al., 2013; Vitor-Costa Okuno et al., 2015). A neural pathway that connects a large number of brain areas, including, the spinal cord, thalamus, secondary somatosensory cortex, medial IC, posterior cingulate cortex, anterior cingulate cortex, premotor area, supplementary motor area, and M1 represents the inhibitory network which leads to fatigue. The balance between inhibitory and facilitatory mechanisms in the M1 optimize the cortical excitability and therefore increase the magnitude of EP. Application of tDCS may induces facilitatory effects to increase motor output from the M1 helps to overcome the existing central fatigue (Vitor-Costa Okuno et al., 2015).

### Reduction of Psychological or Mental Fatigue

Decision making-process during pacing and cognitive control necessary to choose an optimal pacing strategy may be disrupted by mental fatigue (Martin et al., 2018). This disruption is much more evident in long-term aerobic types of exercise. TDCS of DLPFC is a non-invasive technique for the reduction of mental fatigue and therefore enhancement of PE (Nikooharf Salehi et al., 2021). It should be noted that this reduction is only affecting submaximal endurance type of exercise. Literature indicates that mental fatigue does not affect athletes' maximal strength and anaerobic work (Boksem et al., 2006; Dantzer et al., 2014; Martin et al., 2015; Van Cutsem et al., 2017). The difference in the adaptation of athletes for their power output during these long and short duration tasks could be the reason behind this difference (Gandevia, 2001).

### Reduction of RPE

Reduction of RPE following application of tDCS is considered as one of the other reasons behind increased EP in several studies (Okano et al., 2015; Angius et al., 2016a,b). Modulation of sensory perception of effort plays a crucial role in the control of motor output commands (Okano et al., 2015). Overall, the amount of motor commands from M1 or premotor area is considered as the reason behind the changes in RPE (de Morree et al., 2012, 2014; Goodall et al., 2013; Takarada et al., 2014; Zénon et al., 2015).

### Modulation of Autonomic Nervous System Activity

Literature indicates that the autonomic nervous system (ANS) has an important role in the regulation of EP (Okano et al., 2015). Literature supports the association between TC and IC, with ANS control. Therefore, tDCS can modulate the cortical areas directly under the electrodes related to ANS.

ANS is highly related to the mechanisms behind EP and fatigue. It controls homeostatic mechanisms (Damasio et al., 2000; Craig, 2003), especially during PE which requires high metabolic demands (Tulppo et al., 1998; Williamson, 2010). Indeed, ANS responses are linked to EP in healthy individuals (Tanaka et al., 2009). Individuals with higher fitness levels (high aerobic capacity) usually have significantly greater heart rate variability, which is controlled by vagal modulation of the heart rate, compared to individuals with lower fitness levels (low aerobic capacity) (Tulppo et al., 1998).

### Reduction of Perceived Pain

The effects of pain-inducing substances suggest that perception of pain is one of the important regulators of exertion level during fatiguing exercise. Literature shows that a common analgesic such as Acetaminophen increases cycling performance (Mauger et al., 2010). Recent literature indicates that endogenous inhibitory responses, which normally act to decrease nociceptive input and reduce the perception of pain, could be increased following application of tDCS over M1 (Flood et al., 2016). TDCS stimulates descending regions associated with endogenous pain inhibition, enhancing central pain inhibitory responses and causing widespread analgesia (Flood et al., 2016). The association between the pain inhibitory networks and regulation of EP is challenged by the study of Flood et al. (2017) which did not show any significant increase in maximal force production or muscular endurance following application of a single session of high definition tDCS.

## Suggestions for Future Research

There are a number of issues regarding the studies reported in this review which may have implications for future research:

1. Almost all studies reported in this review used single session tDCS. Multiple session tDCS studies are recommended because of its accumulating effects.
2. Majority of studies, used unilateral tDCS of M1. New studies using bilateral tDCS of M1 for both upper limbs, trunk and lower limb muscles are recommended specially in cases that the activity involve trunk and all extremities.
3. Single site tDCS of a brain site was used in almost all of the included studies. It should be noted that multiple sites of the brain never working in isolation. Multi-site application of tDCS is recommended for future studies.
4. In almost all of the included studies, large tDCS electrodes (5 x 7 or 4–6 cm^2^) were used for modulation of single sites of the brain. New tDCS studies using small electrodes is recommended to increase focality of the effects.

In summary, different mechanisms such as facilitation of M1 which causes enhancement of corticospinal excitability, reduction of the supraspinal fatigue, reduction of the psychological/mental fatigue, reduction of PRE, modulation of ANS activity, and reduction of perceived pain play important roles in the enhancement of EP. Literature indicates that modulation of M1, DLPFC, TC, IC, and SMA using unilateral or bilateral tDCS techniques enables us to benefit from these mechanisms.

## Author Contributions

SJ and MZ contributed to all steps including the design of the review to writing of the first and other drafts of the review. Both authors contributed to the article and approved the submitted version.

## Conflict of Interest

The authors declare that the research was conducted in the absence of any commercial or financial relationships that could be construed as a potential conflict of interest.

## Publisher's Note

All claims expressed in this article are solely those of the authors and do not necessarily represent those of their affiliated organizations, or those of the publisher, the editors and the reviewers. Any product that may be evaluated in this article, or claim that may be made by its manufacturer, is not guaranteed or endorsed by the publisher.
